# Corrigendum to: COVID-19 vaccination uptake amongst ethnic minority communities in England: a linked study exploring the drivers of differential vaccination rates

**DOI:** 10.1093/pubmed/fdac021

**Published:** 2022-02-02

**Authors:** Charlotte Hannah Gaughan, Cameron Razieh, Kamlesh Khunti, Amitava Banerjee, Yogini V Chudasama, Melanie J Davies, Ted Dolby, Clare L Gillies, Claire Lawson, Evgeny M Mirkes, Jasper Morgan, Karen Tingay, Francesco Zaccardi, Thomas Yates, Vahe Nafilyan

**Affiliations:** Office for National Statistics, Newport NP10 8XG, UK; Diabetes Research Centre, University of Leicester, Leicester General Hospital, Leicester LE5 4PW, UK; National Institute for Health Research (NIHR) Leicester Biomedical Research Centre (BRC), Leicester General Hospital, Leicester LE5 4PW, UK; Leicester Real World Evidence Unit, Diabetes Research Centre, University of Leicester, Leicester LE5 4PW, UK; Diabetes Research Centre, University of Leicester, Leicester General Hospital, Leicester LE5 4PW, UK; Leicester Real World Evidence Unit, Diabetes Research Centre, University of Leicester, Leicester LE5 4PW, UK; NIHR Applied Research Collaboration – East Midlands (ARC-EM), Leicester General Hospital, Leicester LE5 4PW, UK; Leicester Diabetes Centre, University Hospitals of Leicester NHS Trust, Leicester General Hospital, Leicester LE5 4PW, UK; Institute of Health Informatics, University College London, London NW1 2DA, UK; Department of Cardiology, University College London Hospitals NHS Foundation Trust, London NW1 2PG, UK; Department of Cardiology, Barts Health NHS Trust, London E1 1BB, UK; Diabetes Research Centre, University of Leicester, Leicester General Hospital, Leicester LE5 4PW, UK; Leicester Real World Evidence Unit, Diabetes Research Centre, University of Leicester, Leicester LE5 4PW, UK; NIHR Applied Research Collaboration – East Midlands (ARC-EM), Leicester General Hospital, Leicester LE5 4PW, UK; Leicester Diabetes Centre, University Hospitals of Leicester NHS Trust, Leicester General Hospital, Leicester LE5 4PW, UK; Diabetes Research Centre, University of Leicester, Leicester General Hospital, Leicester LE5 4PW, UK; National Institute for Health Research (NIHR) Leicester Biomedical Research Centre (BRC), Leicester General Hospital, Leicester LE5 4PW, UK; Leicester Diabetes Centre, University Hospitals of Leicester NHS Trust, Leicester General Hospital, Leicester LE5 4PW, UK; Office for National Statistics, Newport NP10 8XG, UK; Diabetes Research Centre, University of Leicester, Leicester General Hospital, Leicester LE5 4PW, UK; Leicester Real World Evidence Unit, Diabetes Research Centre, University of Leicester, Leicester LE5 4PW, UK; NIHR Applied Research Collaboration – East Midlands (ARC-EM), Leicester General Hospital, Leicester LE5 4PW, UK; Leicester Diabetes Centre, University Hospitals of Leicester NHS Trust, Leicester General Hospital, Leicester LE5 4PW, UK; Leicester Real World Evidence Unit, Diabetes Research Centre, University of Leicester, Leicester LE5 4PW, UK; Department of Cardiovascular Sciences, University of Leicester, Leicester LE3 9QP, UK; School of Computing and Mathematical Science, University of Leicester, Leicester LE1 7RH, UK; Office for National Statistics, Newport NP10 8XG, UK; Office for National Statistics, Newport NP10 8XG, UK; Diabetes Research Centre, University of Leicester, Leicester General Hospital, Leicester LE5 4PW, UK; Leicester Real World Evidence Unit, Diabetes Research Centre, University of Leicester, Leicester LE5 4PW, UK; Diabetes Research Centre, University of Leicester, Leicester General Hospital, Leicester LE5 4PW, UK; National Institute for Health Research (NIHR) Leicester Biomedical Research Centre (BRC), Leicester General Hospital, Leicester LE5 4PW, UK; Office for National Statistics, Newport NP10 8XG, UK; Faculty of Public Health, Environment and Society, London School of Hygiene and Tropical Medicine, London WC1E 7HT, UK

In the originally published version of this manuscript, incorrect images were included for Figures 1 and 2 in error. These errors have been corrected. The figure legends are unchanged.

